# Primary carcinoma of the larynx in females: A case series

**DOI:** 10.1016/j.amsu.2022.103851

**Published:** 2022-05-25

**Authors:** Y. Oukessou, A. Chebaatha, O. Berrada, R.L. Abada, S. Rouadi, M. Roubal, M. Mahtar, M. Regragui, M. Karkouri

**Affiliations:** a ENT Head and Neck Surgery Department, Ibn Rochd University Hospital, Faculty of Medicine and Pharmacy, Hassan II University, Casablanca, Morocco; b Pathology Department, Ibn Rochd University Hospital, Faculty of Medicine and Pharmacy, Hassan II University, Casablanca, Morocco

**Keywords:** Larynx, Laryngeal carcinoma, Women, Female, Cancer

## Abstract

**Background and aim:**

In north Africa, laryngeal carcinomas remain a predominately male pathology. While in many countries the gap between men and women is narrowing. This study aimed to examine the epidemiological, clinical, therapeutic, and follow up data of a case series of 23 female patients treated for laryngeal carcinoma.

**Patients and methods:**

Medical records of a case series of 23 patients for primary carcinoma of the larynx at the Department of Head and Neck Surgery of the 20 August Hospital of Casablanca, between January 2012 and September 2016, were reviewed. Demographic, clinical, endoscopic, radiological, surgical, and follow-up data were collected.

**Results:**

7% of all the patients treated for LC were women, The most affected age group was between 60 and 79 years (52%), 52% had no major risk factor, all patients had an epidermoid carcinoma, 48% of patients had T2 tumors. T1, T3, and T4a were found in respectively 17%, 22%, and 13%. N1 in 43% of the cases (n = 10), N0 in 35% (n = 8), N2b in 17% (n = 4), N2c in 4% (n = 1). All patients were M0. All the patients in this series have undergone surgical treatment. At 5 years, the survival rate was 83%.

**Conclusion:**

Since the proportions of women in published studies are limited, there are still many controversies about gender differences in laryngeal cancer. Therefore, further studies should seek a clearer understanding of factors involved in female laryngeal cancer to adopt more appropriately the measures of prevention and early diagnosis.

## Highlights

• Larynx cancer is among the neoplasms with greatest gender differences found in most populations worldwide.
• Controversies and limited studies about gender differences in laryngeal cancer.
• Many studies emphasize that its impact seems to be higher for female patients as opposed to males.

## 1 Introduction

**Larynx carcinomas affect generally men in the fifth and sixth decades.** The incidence rate of laryngeal cancer (LC) shows considerable geographical variability in both males and females [1]. **In Morocco, it represents the most frequent ENT cancer that counts for 2% of all cancers, and it's the eighth most common cancer in men (4.1% of all cancers). In women, LC is still rare, its incidence is estimated at 0.4% (7,7% of LC). In western countries, the gap of incidence and death between men and women has been narrowing during the past 3 decades** [2]

Consumption of tobacco and alcohol are the main risk factors for laryngeal cancer, along with occupational exposure to industrial carcinogenic substances. Other risk factors are identified in the scientific literature, but with less conclusive evidence [3].

This descriptive study aims to present the results of an analysis of a clinical series of 23 Moroccan female patients with cancer of the larynx treated with surgery alone or a combination of surgery and postoperative chemoradiotherapy, in a Tertiary ENT referral center in Casablanca, Morocco. To date, only a few papers describing this entity in the north African area are available. This work is reported in line with the PROCESS 2020 criteria [4].

## 2 Patients and Methods

### 2.1 Study design

This was a retrospective study (case series) of 23 patients treated by an ENT Professor for primary carcinoma of the larynx at the Department of Head and Neck Surgery of the 20 August Hospital of Casablanca, between January 2012 and September 2016.

### 2.2 Study criteria

We excluded from the study the patients who refused any treatment as well as all the incomplete files, patients having histological types other than carcinomas or cancers of adjacent structures invading the larynx.

### 2.3 Data collection

A database was created using the surgical records of the patients.

The following data were recorded for each patient: Demographic, clinical, endoscopic, radiological, surgical, and follow-up.

### 2.4 Setting

Data were collected on forms, and were computerized and analyzed using IBM SPSS Statistical Software: Release 25. Continuous variables are presented as the mean (± standard deviation) and categorical variables are presented as counts.

### 2.5 Registration and ethics

All subjects or their parents were informed about the retrospective nature of the study and provided their consent before surgery. According to Moroccan laws. We did not seek approval from the ethics committees of the service as it is a retrospective study based on chart analysis. No human or animal experiments were involved in our study. This is a case series that does not require a research registry.

## 3 Results

**Fig. 1:**
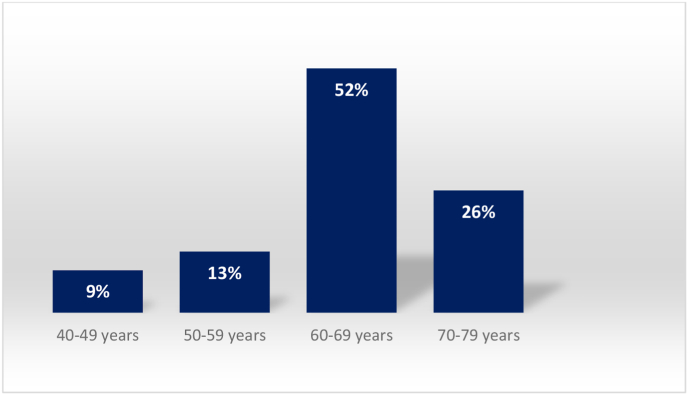
Age distribution.

Between January 2012 and September 2016, 328 patients with larynx carcinoma were operated on at the ENT/Head and Neck surgery department of the Ibn Rochd university hospital, Casablanca, Morocco. 7% of the patients were female (n = 23). The median age was 64 years (range 47–79 years). The most affected age group was between 60 and 79 years, it represented 52% of all patients [Fig. 1].

In our series, 21 patients (91%) benefited from a family environment, whereas two patients were socially isolated (9%).

Ten of the patients (43%) were smokers (Fig. 3). Three of them were passive and the other seven were active smokers (30%) with an average consumption of 18.5 pack-years. Yellow industrial tobacco was the main mode of consumption.

**Fig. 2:**
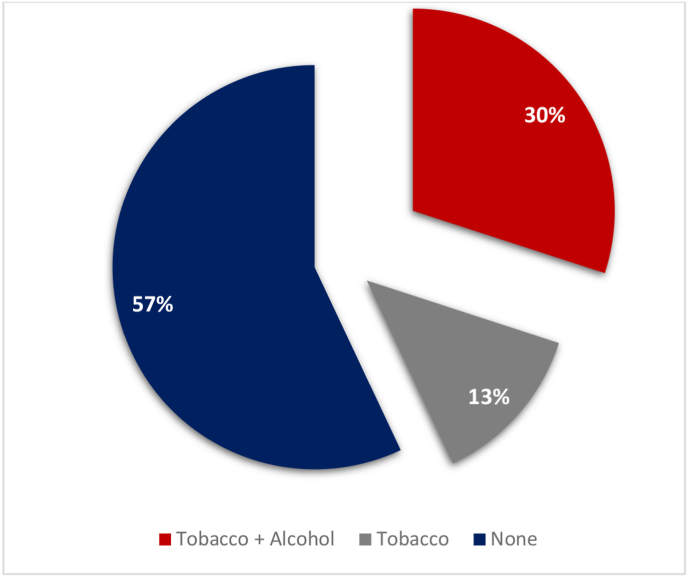
Smoking and alcohol consumption data.

Alcohol use was found in 7 people (30%), and all of them were active smokers [Fig. 2].

**Fig. 3:**
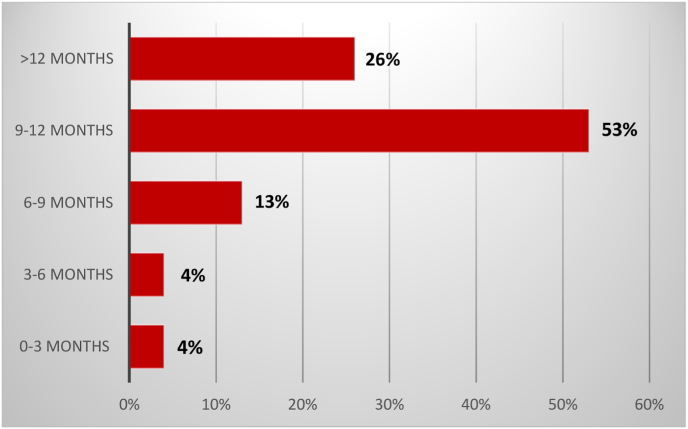
Lag time between the onset of symptoms and first consultation.

19 patients (82%) had poor oral health with numerous untreated dental caries. GERD symptoms were present in five patients (22%). The notion of exposure to wood oven smoke was found in five patients (22%), all from rural areas.

Exposure to other chemicals (cement, asbestos, chromium, nickel) incriminated in laryngeal oncogenesis was not specified in the files. All of our patients were housewives and were of low socioeconomic status.

No patient had a history of cervical irradiation nor a family history of laryngeal cancer.

The mean lag time between the onset of symptoms and the first consultation was 11 months (6 months–5 years) **[**Fig. 3**].**

**Fig. 4:**
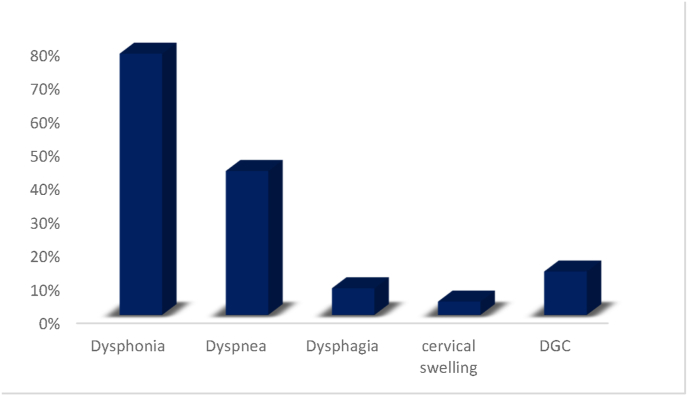
Distribution of patients according to symptoms of discovery.

Dysphonia was the leading revealing symptom (78%, n = 18), that was associated with dyspnea in 43% of cases. Dysphagia was seen in only 2 cases (8%). Cervical swelling was found in one case (4%). Three patients (13%) experienced a deterioration of general condition [Fig. 4].

All the patients underwent a clinical examination, a CT scan, and a laryngoscopy under general anesthesia with biopsy.

The epidermoid carcinoma was well, moderately and poorly differentiated in respectively 78% (n = 18), 13% (n = 3) and 11% (n = 2) 18. No Patient had a synchronous cancer. The tumor localizations were as follows: glottic in 17% (n = 4), supra-glottic in 13% (n = 3), Glotto-supraglottic in 30% (n = 7), Glotto-infraglottic in 17% (n = 4), and Glottic, supra and infraglottic in 22% (n = 5).

According to the UICC 2017 classification, 48% of patients had T2 tumors. T1, T3, and T4a were found in respectively 17%, 22%, and 13%. No patient had a T4b stage **[**Tab le1**].** The Lymph node assessment showed N1 in 43% of the cases (n = 10), N0 in 35% (n = 8), N2b in 17% (n = 4), N2c in 4% (n = 1). No patient presented an N2a or N3 cervical lymph node metastasis **[**Tab le2**].** All patients were M0. Table 3 summarizes the distribution of patients according to tumor stage **[**Tab le3**].**

**Table 1:** T classification according to UICC 2017.

Classification	Pourcentage
T1	17%
T2	48%
T3	22%
T4	T4a: 13; T4b: 0%

**Table 2:** N classification according to UICC 2017.

Classification	Pourcentage
N0	35%
N1	43%
N2	N2a: 0%; N2b:17%; N2c:4%
N3	0%

**Table 3:** Distribution of patients according to tumor stage.

Stages	Pourcentage/Numbers of patients
**Stage I**	**17% - n = 4**
T1N0M0	n = 4
**Stage II**	**8% - n = 2**
T2N0M0	n = 2
**Stage III**	**39% - n = 9**
T2N1M0	n = 8
T3N1M0	n = 1
**Stage IV**	**35% - n = 8**
T2N2cM0	n = 1
T3N2bM0	n = 4
T4N0M0	n = 2
T4N1M0	n = 1

All the patients of this series have undergone a surgical treatment performed by ENT Professor. 17% of patients (n = 4, T1) were treated with an endoscopic laser cordectomy. 35% (n = 8, T3, and T4) with a total laryngectomy associated with postoperative radiotherapy. 48% (n = 11, T2) with a supra-cricoid laryngectomy. Bilateral levels II to IV neck dissection were performed for T2 tumors and above. Excision limits were safe except for 1 case of supracricoid laryngectomy that needed a totalization (4%).

The mean duration of hospital stay was 8.42 days (5-14). 2 patients presented a wound site infection after total laryngectomy complicated with pharyngocutaneous fistula that has been successfully managed with local care and antibiotherapy. These complications didn't lead to any delay in postoperative radiotherapy. No major complications were noted.

At 12 months follow-up, 2 patients initially treated with supracricoid laryngectomy had presented a local recurrence. A totalization was performed for one, and a radiochemotherapy for the other.

At 5 years, the survival rate was 83%. All the patients have beneficiated from vocal and swallowing rehabilitation.

## 4 Discussion

Squamous cell carcinomas (SCC) of the larynx are the most common malignancies of the upper aerodigestive tract in the Western hemisphere. They occur with an incidence of about 5–7 cases per 100,000 individuals each year, mainly affecting men between the ages of 55 and 65 years. The male-to-female ratio has been determined to range between 20 and 1 [6] and 32 to 1 [5].

Larynx cancer is among the neoplasms with the greatest gender differences found in most populations worldwide [6]; In Morocco, the 2008–2012 Cancer registry of Grand Casablanca reported a 0.4 per 100,000 person-years incidences of laryngeal cancer in females among the Moroccan population versus 4.6 for men. This large gap in incidence according to gender is very likely to be due to the difference in levels of tobacco and alcohol exposure. In 2017, the incidence of smoking was 74.75 times higher among men than women, whereas alcohol consumption was as low as 0.4% for women vs 7.1% for men [7]. Since the LC incidence stayed stable, the trend of the gap narrowing incidence and mortality of LC between men and women, as reported in many western countries (such as the USA, England, and Wales) [8] has not been observed in Morocco nor in neighboring countries [9].

In this series, 57% of patients have never smoked or consumed alcohol. These findings might indicate that laryngeal cancer in our context is probably linked to factors other than smoking or alcoholism.

The large male to female ratio has led to the suggestions that they might be other factors influencing the development of carcinomas such as hormonal influences, genetic predisposition, and others.

The larynx has been considered a secondary sex organ that undergoes developmental and physiologic changes during puberty and throughout the menstrual cycle, combined with the high rate of laryngeal carcinomas in men, has led to the suggestion that laryngeal carcinoma might be a sex hormone-dependent tumor similar to prostate and mammary carcinoma [5]**.**

Chaudhri et al. found that there is compelling evidence that laryngeal cancer is hormone-responsive, specifically to the effects of E2, via ERα36. This membrane receptor activates several pathways, which were found both in vitro and in vivo, to correlate with laryngeal cancer progression and aggressiveness [10]**.**

However, Hagedorn et al. strongly argue against laryngeal carcinoma being sex-hormone-dependent tumors because they couldn't find any significant amount of male and female sex-hormone receptors in an immunomorphological and biochemical study on both in-vivo and in-vitro tumor cells [5]**.**

HPV infection, especially infection due to the high-risk type HPV-16, was found to be significantly associated with the risk of laryngeal squamous cell carcinoma [11].

It may have a synergistic effect with tobacco use, the viral cell replication may amplify the mutagenesis and metaplasia caused by smoking [12]**.**

GERD and poor oral health are also biologically plausible risk factors for laryngopharyngeal cancers that were respectively present in 22% and 82% of the patients [13,14]**.**

In some series analyzing gender differences in LC, it was reported that women presented with a higher degree of supraglottic involvement, whereas men predominantly presented with glottic carcinomas [15]. The reason for this difference in subsite involvement is not clear yet. As patterns of tobacco smoking and alcohol consumption change over time, say, with a decline in the proportion of male smokers and a rise in female smokers, we might expect to see both the incidence rate ratio, and the distribution of anatomical subsites equalize [8]. In our series of female patients, the glottis was more frequently involved than the supra-glottis.

Gender differences in prognosis are debatable, according to some authors, female patients with laryngeal carcinoma have a better chance of survival than males, especially when the initial tumors are diagnosed in the early stages. For others, no difference was found [16,17].

Kokoska et al. reported in a multivariable analysis that the 5-years survival rate for women was linked to age, comorbidity, and severity of symptoms, while it was linked to age, comorbidity, and anatomic subsite [18].

Treatment of laryngeal cancer in women is the same as in males. After total laryngectomy (TL), alaryngeal speech is very challenging for the speaker, which can result in a high degree of voice handicap. For example, an acoustic analysis by Kazi et al. found that gender frequency differences are lost following TL for tracheoesophageal speakers. Furthermore, alaryngeal voice affected the confidence of women more than men and suffer from more concerns related to body image [19].

Such concerns also may be quite applicable to those who receive less radical (conservative) treatment methods. That is, in some instances, partial laryngectomy may result in relatively greater levels of disability for a female as opposed to a male patient (e.g., a "rough'" voice quality may garner more social penalty in a woman) [20].

Katz et al. have studied psychosocial impact after head and neck cancer surgical treatment, they demonstrated that women seemed to be more vulnerable, with higher levels of depression and lower life happiness; their adjustment was more closely linked to their social environment [21].

## 5 Conclusion

Since the proportions of women in published studies are limited, there are still many controversies about gender differences in laryngeal cancer. Therefore, further studies should seek a clearer understanding of etiopathogenic factors involved in female laryngeal cancer, especially in those without any significant smoking or alcohol exposure. There should also be an increased awareness among medical practitioners and the general population that laryngeal carcinoma is not a disease affecting exclusively heavy smokers and drinkers. Many studies emphasize that its impact seems to be higher for female patients as opposed to males, hence the importance of prevention and early detection.

## Ethical approval

I certify that this kind of manuscript does not require ethical approval by the Ethical Committee of our institution.

## Source of funding

This research did not receive any specific grant(s) from funding agencies in the public, commercial, on non-for-profit sectors.

## Author contributions

Y. Oukessou: writing the paper, study concept.

A. Chebaatha: Corresponding author.

O. Berrada: acquisition of data.

S. Rouadi: revising the article.

R. Abada: revising the article.

M. Roubal: revising the article.

M. Mahtar: final approval of the version to be submitted.

M.Regragui: study concept.

M. Karkouri: study concept.

## Registration of research studies

This is a cohort study that does not require a research registry.

## Guarantor

The Guarantor is the one or more people who accept full responsibility for the work and/or the conduct of the study, had access to the data, and controlled the decision to publish.

## Consent

Written informed consent was obtained from the patients for publication of this cohort study and accompanying images. A copy of the written consent is available for review by the Editor-in-Chief of this journal on request.

## Declaration of competing interest

The authors declare that they have no competing interests.
